# Dityrosine-Lowering *Lactiplantibacillus plantarum* HNGD-XJ9: Screening, Characterization, and Evaluation

**DOI:** 10.3390/foods15213778

**Published:** 2026-10-22

**Authors:** Yueting Ge, Kaile Chen, Zhenguo Tian, Hao Zheng, Zihao Jin, Chengjia Gu, Guowei Le, Yuhui Yang

**Affiliations:** 1 Henan Key Laboratory of Tea Plant Biology, College of Tea and Food Science, Xinyang Normal University, Xinyang 464000, China; ytge@xynu.edu.cn (Y.G.);; 2 College of Food Science and Engineering, Henan University of Technology, 100 Lotus Street, Hi-Tech Development District, Zhengzhou 450001, China; 3 State Key Laboratory of Food Science and Technology, School of Food Science and Technology, Jiangnan University, Wuxi 214122, China

**Keywords:** dityrosine, *Lactiplantibacillus plantarum*, protein oxidation, lactic acid bacteria, screening

## Abstract

Dityrosine, a specific protein oxidation product formed through oxidative crosslinking of tyrosine residues, accumulates in foods and is associated with potential health risks. The removal of pre-existing dityrosine from food matrices remains a significant challenge. Given the metabolic versatility of lactic acid bacteria (LAB) in amino acid catabolism, they may provide a promising biological solution for this problem. In the present work, a food-grade LAB with high dityrosine-lowering activity was screened from naturally fermented food isolates through targeted screening. Among 1213 presumptive LAB strains, HNGD-XJ9 exhibited the highest reduction efficiency and was identified as *Lactiplantibacillus plantarum* by phylogenetic, morphological, and whole-genome ANI analyses. The strain exhibited favorable gastrointestinal tolerance, notable adhesive properties, and no hemolytic activity, indicating its in vitro probiotic-related properties. Whole-genome sequencing revealed candidate genes involved in aromatic amino acid metabolism and aromatic ring cleavage, providing molecular insights into its putative mechanism for dityrosine reduction and offering genomic clues for further investigation of its probiotic-related properties. Fermentation with HNGD-XJ9 significantly reduced detectable dityrosine levels by 70.89% in spray-dried milk powder and 58.61% in roasted pork powder (*p* < 0.05), along with decreases in other oxidative markers and partial restoration of protein conformational integrity. These results suggest that HNGD-XJ9 is a promising strain for mitigating dityrosine accumulation in foods and for developing functional foods with lower protein oxidation-related risks.

## 1. Introduction

Dityrosine, a stable dimeric fluorescent derivative formed through free radical-mediated oxidative crosslinking of tyrosine residues, is widely recognized as a reliable and specific biomarker of oxidative damage to protein in foods [1,2]. Its formation occurs under various conditions commonly encountered during food processing and storage, including thermal treatment, irradiation, mechanical stress, and exposure to light or metal ions, with the extent of dityrosine accumulation being positively correlated with processing intensity [3,4,5,6]. Mechanistically, these conditions promote the production of reactive oxygen species (ROS) and other free radicals, which oxidize tyrosine residues to tyrosyl radicals; subsequent radical–radical coupling yields dityrosine [1]. During storage, oxygen, light, and lipid oxidation products can further promote this radical-mediated crosslinking [7]. As a prevalent protein oxidation product in thermally processed foods, the excessive accumulation of dityrosine disrupts the native structural properties of food proteins, leading to protein aggregation [8,9] and impaired functionality in various food systems, such as clotting in dairy products and textural hardening in meat products, thereby significantly diminishing nutritional quality and commercial value [10,11,12]. More critically, dietary intake of dityrosine contributes to its sustained accumulation in the human body, where it has been implicated in the disruption of intestinal barrier integrity, induction of oxidative stress and chronic inflammation, and association with various chronic diseases including Alzheimer’s disease and metabolic syndrome [13,14,15]. Consequently, the development of safe and efficient approaches to lower dityrosine levels in foods has emerged as a critical concern for food nutrition and safety.

Over the past few years, lactic acid bacteria (LAB) have drawn increasing attention for their ability to modulate food quality through metabolic activities during fermentation [16,17]. LAB are known to possess a rich repertoire of enzymes that participate in protein hydrolysis and amino acid metabolism, enabling them to degrade macromolecular proteins into peptides and free amino acids, and further metabolize amino acids through various catabolic pathways [18]. For instance, fermentation with various LAB strains has been shown to significantly modify the nutritional composition of food substrates, including changes in protein, free amino acid, and phenolic content [19]. Notably, certain LAB strains have been shown to influence protein oxidation processes in food matrices. For instance, *Lactobacillus plantarum* NJAU-01 markedly reduced protein carbonyl levels and surface hydrophobicity while increasing sulfhydryl levels during sausage fermentation [20], indicating its capacity to mitigate oxidative modifications of proteins. Similarly, fermentation with *Lactobacillus sakei* or *L. plantarum* has been reported to affect protein hydrolysis and oxidation patterns in meat products [21]. Moreover, LAB have demonstrated the ability to degrade various food-borne hazardous compounds through enzymatic hydrolysis and metabolic transformation [22,23,24]. Additionally, certain probiotic strains have been reported to alleviate oxidative stress and neutralize reactive carbonyl species, which may indirectly contribute to lowering the levels of advanced glycation end products (AGEs), another class of protein oxidation products [25]. The ability of LAB to metabolize aromatic amino acids has also been documented; for example, *Lactococcus lactis* possesses aromatic aminotransferases that initiate the catabolism of aromatic amino acids, including tyrosine, to various metabolites [26]. Tyrosine metabolism pathways have been identified in multiple *Lactobacillus* and *Lactiplantibacillus* species, as documented in Kyoto Encyclopedia of Genes and Genomes (KEGG) pathway databases, and *Lactobacillus plantarum* has been documented to convert tyrosine to tyramine [27]. Additionally, *Lactobacillus gasseri* has been shown to suppress the transformation of tyrosine into p-cresol in the colon [28]. These findings collectively suggest that LAB harbor the enzymatic machinery necessary for metabolizing tyrosine and its derivatives, raising the possibility that certain LAB strains may be capable of reducing dityrosine, a stable crosslinked dimer of tyrosine, in food matrices. However, as far as we know, no LAB strain has been documented to efficiently reduce pre-existing dityrosine in foods, and the molecular mechanisms underlying such reduction remain unexplored.

Based on these observations, we hypothesized that certain LAB strains with the ability to reduce dityrosine may exist in naturally fermented foods. To test this hypothesis, a previously established collection of 1213 presumptive LAB isolates derived from naturally fermented foods [29] was screened for dityrosine-lowering activity. This work aimed to select the most efficient dityrosine-lowering strain from this collection and to comprehensively characterize it, including taxonomic identification, evaluation of in vitro probiotic-related properties and hemolytic activity, assessment of efficacy in reducing detectable dityrosine in real food matrices, and elucidation of the putative molecular mechanisms via whole-genome analysis. As far as we know, no previous study has reported the identification and characterization of a LAB strain capable of reducing dityrosine in foods. The findings are expected to provide a novel biological strategy for mitigating dityrosine accumulation in processed foods and to provide a theoretical foundation for developing functional products with reduced protein oxidation-related risks.

## 2. Materials and Methods

### 2.1. Sample Preparation

The 1213 presumptive LAB isolates used in this study were obtained from a previously established collection, as described in our previous study [29]. This collection originated from 370 samples of naturally fermented foods obtained from different regions of China. The isolates were preliminarily identified as presumptive LAB based on the following criteria: growth on MRS agar supplemented with 0.04% (*w*/*v*) bromocresol purple, formation of yellow clear zones, Gram-positive staining, and catalase-negative reaction. Detailed isolation and purification procedures have been described previously [29]. These isolates were stored in MRS broth containing 20% glycerol at −80 °C and activated in MRS broth before screening.

Pasteurized milk and fresh pork tenderloin were used as the raw materials. The preparation of low- and high-oxidation milk and pork powder samples was performed as described in our previous study [29]. Briefly, low-oxidation milk powder was obtained by freeze-drying, while high-oxidation milk powder was produced by spray-drying (180 °C inlet, 90 °C outlet). For pork, low-oxidation powder was prepared by sous-vide cooking (65 °C, 10 h), and high-oxidation powder was prepared by oven roasting (180 °C, 30 min). All samples were stored at −80 °C.

### 2.2. Reagent Preparation

Dityrosine selective medium was prepared by dissolving 5.0 g/L sodium acetate, 20.0 g/L glucose, 2.0 g/L K_2_HPO_4_, 2.0 g/L diammonium citrate, 0.2 g/L MgSO_4_·7H_2_O, 0.04 g/L MnSO_4_·H_2_O, and 1.0 mL/L Tween 80 in distilled water, supplemented with 0.5 g/L dityrosine (purity ≥ 98%, Henan Wanbang Chemical Technology Co., Ltd., Zhengzhou, China) as the primary nitrogen source. All media and buffers were sterilized by autoclaving (121 °C, 20 min). All reagents used were of analytical purity.

### 2.3. Screening of LAB for Dityrosine-Lowering Activity

The preserved isolates were revived in MRS broth (37 °C, 24 h). For primary screening, the cultures were harvested by centrifugation, rinsed twice with sterile saline, resuspended to an OD_600_ of 1.0, and then inoculated (2%, *v/v*) into the dityrosine selective medium, followed by incubation at 37 °C for 72 h. Following incubation, supernatants were collected by centrifugation. The residual dityrosine content in the supernatant was determined by fluorescence spectroscopy at excitation and emission wavelengths of 325 nm and 400 nm, respectively, according to the method of Xiong et al. [30]. The reduction rate of dityrosine was then calculated as Reduction rate (%) = (F_0_ − F_1_)/F_0_ × 100%, where F_0_ and F_1_ are the fluorescence intensity of the blank control (uninoculated medium) and the sample supernatant, respectively. Candidates showing reduction rates above 50% in the primary screening proceeded to secondary screening.

During the secondary screening, the selected strains were re-activated in MRS broth to an OD_600_ of 1.0, then inoculated at a 2% (*v/v*) ratio into fresh MRS broth containing dityrosine (0.5 g/L) and maintained at 37 °C for 24 h. Following centrifugation, the supernatants were collected and the residual dityrosine content was determined using the same method. The strain exhibiting the greatest and most stable reduction activity was chosen for further characterization.

### 2.4. In Vitro Probiotic-Related Properties and Hemolytic Activity

The candidate strains were tested for their tolerance to acid and bile salts using the protocol of Gupta et al. [31]. Briefly, artificial gastric juice (pH 2.0 or 3.0) and artificial intestinal juice (pH 7.5) containing 0.15% or 0.3% (*w*/*v*) ox bile salt were prepared. Activated strains (~10^8^ CFU/mL) were inoculated into artificial gastric or intestinal juice (1:9, *v/v*) and incubated at 37 °C. At 0, 2, and 4 h, samples were plated on MRS agar for viable cell enumeration. The survival rate was calculated as (A_t_/A_0_) × 100%, where A_0_ and A_t_ represent the viable cell counts at 0 h and after 2 or 4 h of incubation, respectively.

Cell surface hydrophobicity and auto-aggregation were measured as described previously [32]. For hydrophobicity, a cell suspension (OD_600_ ≈ 0.5, A_0_) was combined with an equal volume of xylene, agitated for 2 min, and kept at 37 °C for 1 h to allow phase separation. The OD_600_ of the aqueous layer (A_1_) was then recorded. Hydrophobicity (%) = [(A_0_ − A_1_)/A_0_] × 100. For auto-aggregation, the cell suspension (OD_600_ ≈ 0.5, A_0_) was statically incubated at 37 °C, and the OD_600_ of the top layer (A_t_) was recorded after 4 h. Auto-aggregation (%) = [1 − (A_t_/A_0_)] × 100.

Hemolytic activity was examined on Columbia blood agar plates supplemented with 5% defibrinated sheep blood, following the procedure of Ou et al. [33]. The selected strain was streaked onto the plates and cultured anaerobically at 37 °C for 48 h. *Staphylococcus aureus* ATCC 25923 (β-hemolysis) and *Lactiplantibacillus plantarum* ATCC 14917 (γ-hemolysis) were used as the positive and negative references, respectively. Following the incubation period, zones of clearance surrounding the colonies were checked, which are indicative of β-hemolytic activity.

### 2.5. Identification and Physiological Characterization

For molecular identification, genomic DNA extraction, 16S rDNA amplification, sequencing, BLAST+ (version 2.16.0) comparison, and phylogenetic tree construction were carried out as described in our previous study [29]. The universal primers used were 27F (5′-AGAGTTTGATCCTGGCTCAG-3′) and 1492R (5′-GGTTACCTTGTTACGACTT-3′). Species identity was further verified by ANI analysis (EzBioCloud ANI Calculator, OrthoANIu algorithm) between the HNGD-XJ9 genome and the type strain *Lactiplantibacillus plantarum* ATCC 14917^T^ (NCBI RefSeq assembly accession: GCF_001434175.1).

Colony morphology was examined after 24 h of incubation on MRS agar at 37 °C. Gram staining was carried out as described previously [34], and the stained cells were observed microscopically (Olympus CX31 RTSF, Tokyo, Japan). For scanning electron microscopy, cells were prepared and observed as described previously [35] using a scanning electron microscope (Zeiss Sigma 300, Jena, Germany).

The optimal growth temperature and initial pH were determined as described in our previous study [29]. Briefly, the strain was cultured at 2% (*v/v*) in MRS broth at 27–42 °C (5 °C intervals) for 24 h to determine the optimal temperature, and at 37 °C with pH 5.0–8.5 (0.5 intervals) for 24 h to determine the optimal pH, followed by OD_600_ measurement. Growth kinetics and acidification profiles were simultaneously monitored by inoculating the activated strain (2%, *v/v*) into MRS broth at 37 °C and recording OD_600_ and pH every 2 h throughout a 28 h cultivation period.

### 2.6. Whole-Genome Sequencing

HNGD-XJ9 was subjected to whole-genome sequencing by Biomarker Biotechnology Co., Ltd. (Beijing, China) using paired-end sequencing on an Illumina MiSeq system. The raw reads were quality-filtered, assembled de novo, and functionally annotated following the pipeline described in our previous study [29]. Antibiotic resistance and virulence-related genes were initially screened using the same criteria as in [29]. In this study, sequences with ≥80% identity and ≥80% coverage over the query length were further considered positive hits for potential safety concerns, while sequences below these thresholds were considered non-significant.

### 2.7. Evaluation of Dityrosine-Lowering Efficacy in Food Matrices

The spray-dried milk powder and roasted pork powder (prepared according to Section 2.1) were suspended in sterile water at 1:9 and 1:4 (*w/v*) ratios, respectively. Fermentation was initiated by adding the activated HNGD-XJ9 culture (10^8^ CFU/mL) to a final concentration of 2% (*v/v*), and the mixtures were held for 48 h at 37 °C. Following fermentation, the samples were lyophilized and kept at −80 °C until analysis. Unfermented without bacterial inoculation were used as oxidation controls.

The contents of various protein oxidation products were quantified following previously established protocols. Dityrosine was determined by fluorescence spectroscopy [30]. Carbonyl levels were measured by DNPH colorimetry [36]. Schiff bases content was measured via fluorescence spectroscopy with excitation at 350 nm and emission at 420 nm [37]. Kynurenine and N′-formylkynurenine were quantified by fluorescence spectroscopy with excitation/emission pairs of 365/480 nm and 325/434 nm, respectively [38]. Free thiols were assayed using Ellman′s reagent [39], and free amino groups were determined by the o-phthaldialdehyde (OPA) method [40]. Protein content was determined by Kjeldahl method.

### 2.8. Structural and Microstructural Characterization

Microstructural observation was performed using confocal laser scanning microscopy (CLSM). Fluorescein isothiocyanate (FITC) was used to label protein samples, which were then visualized using a confocal laser scanning microscope (OLYMPUS FV3000, Japan) with 488 nm excitation, following the method described previously [41].

Fourier-transform infrared (FTIR) spectroscopy was performed as described in [29]. In brief, freeze-dried samples were ground with spectroscopic-grade potassium bromide (KBr, 1:100, *w/w*) and pressed into pellets. After baseline correction, secondary structure components were calculated from the amide I region (1700–1600 cm^−1^) using OMNIC 8.2 and PeakFit 4.0 [42].

### 2.9. Statistical Analysis

Three independent biological replicates (*n* = 3) were used for all experiments, with triplicate technical measurements per replicate. Data were analyzed using SPSS 27.0 (IBM Corporation, Armonk, NY, USA), and results are expressed as mean ± SEM. Prior to group comparisons, data normality and variance homogeneity were checked by the Shapiro–Wilk test and Levene’s test, respectively. When both assumptions were met, one-way ANOVA with Tukey’s HSD post-hoc test was used for multiple comparisons; if variance homogeneity was violated, Tamhane’s T2 test was applied instead. Statistical significance was set at *p* < 0.05. Graphs were generated with GraphPad Prism 8.0.2 (GraphPad Software, San Diego, CA, USA). In all figures and tables, different lowercase letters (a, b, c) denote significant differences among groups (*p* < 0.05).

## 3. Results and Discussion

### 3.1. Screening of LAB for Dityrosine-Lowering Activity

**Figure 1:**
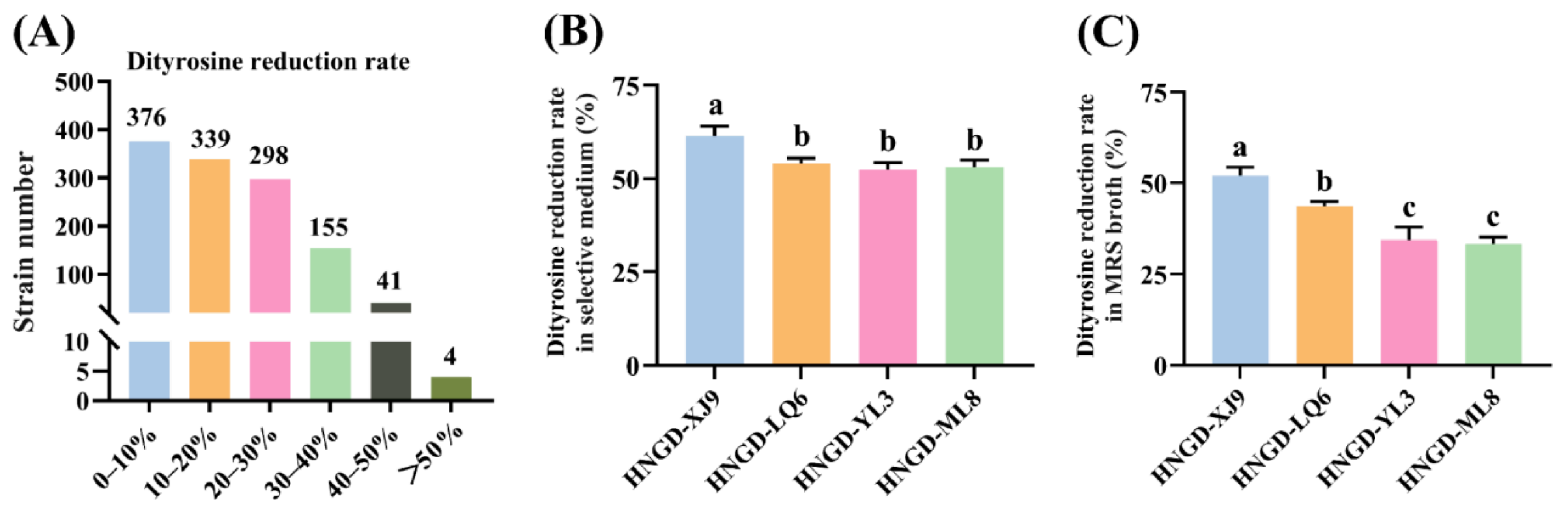
Screening of lactic acid bacteria for dityrosine-lowering activity. (**A**) The 1213 isolates were grouped according to their dityrosine reduction rate in the selective medium. (**B**,**C**) Dityrosine reduction rates of four candidate strains in selective medium (**B**) and MRS broth (**C**). Values are presented as mean ± SEM from three independent biological replicates (*n* = 3). In panels (**B**,**C**), values with different lowercase letters (a, b, c) denote significant difference (*p* < 0.05), whereas identical letter indicates no significant difference.

The 1213 presumptive LAB isolates used in this study were derived from 370 naturally fermented foods obtained from different regions of China, covering a broad spectrum of fermented foods (bean-based, vegetable, fruit, flour, meat, wine, dairy, and tea products), as described in our previous study [29]. These isolates were subjected to a screening process using a dityrosine-supplemented selective liquid medium in which dityrosine served as the main nitrogen source. As shown in Figure 1A, the majority of the isolates exhibited detectable dityrosine-lowering activity, with most strains showing reduction rates below 40%. Only four strains, namely HNGD-XJ9, HNGD-ML8, HNGD-LQ6, and HNGD-YL3, showed reduction rates above 50%. This low proportion of highly efficient strains (approximately 0.33%) is consistent with findings from other functional screening studies targeting specific foodborne substrates [22,23,24], indicating that highly efficient dityrosine-lowering strains are relatively rare in natural ecosystems. For instance, screening of 40 LAB strains for acrylamide removal [22], 10 LAB strains for organophosphorus pesticide degradation [23], and 80 samples for purine nucleoside degradation [24] all yielded only a small number of highly efficient strains, further supporting that highly efficient strains are generally rare in natural microbial communities.

To confirm this finding, the four candidate strains were re-evaluated in the dityrosine selective medium (Figure 1B). All four strains maintained reduction rates above 50%, confirming the reliability of the initial screening, with HNGD-XJ9 showing the highest activity (61.39%), followed in descending order by HNGD-LQ6 (54.06%), HNGD-ML8 (53.10%), and HNGD-YL3 (52.39%) (*p* < 0.05). To further evaluate the stability of their dityrosine-lowering activity under nutritionally complex conditions, a second round of verification was conducted in MRS broth containing added dityrosine, which better simulates the nutrient-rich environment of real food systems. As presented in Figure 1C, HNGD-XJ9 consistently demonstrated a markedly higher reduction rate (52.00%) than the other three strains (*p* < 0.05). The screening approach used here, in which dityrosine served as the main nitrogen source, differs fundamentally from previous strategies that primarily focused on inhibiting dityrosine formation through physical or chemical means [1]. The superior and stable performance of HNGD-XJ9 across both selective and nutrient-rich media suggests that its dityrosine-lowering activity is constitutively expressed, making it a promising candidate for further characterization and application studies.

### 3.2. In Vitro Probiotic-Associated Characteristics and Hemolytic Activity of Candidate Strain

#### 3.2.1. Tolerance to Acid and Bile Salts

**Figure 2:**
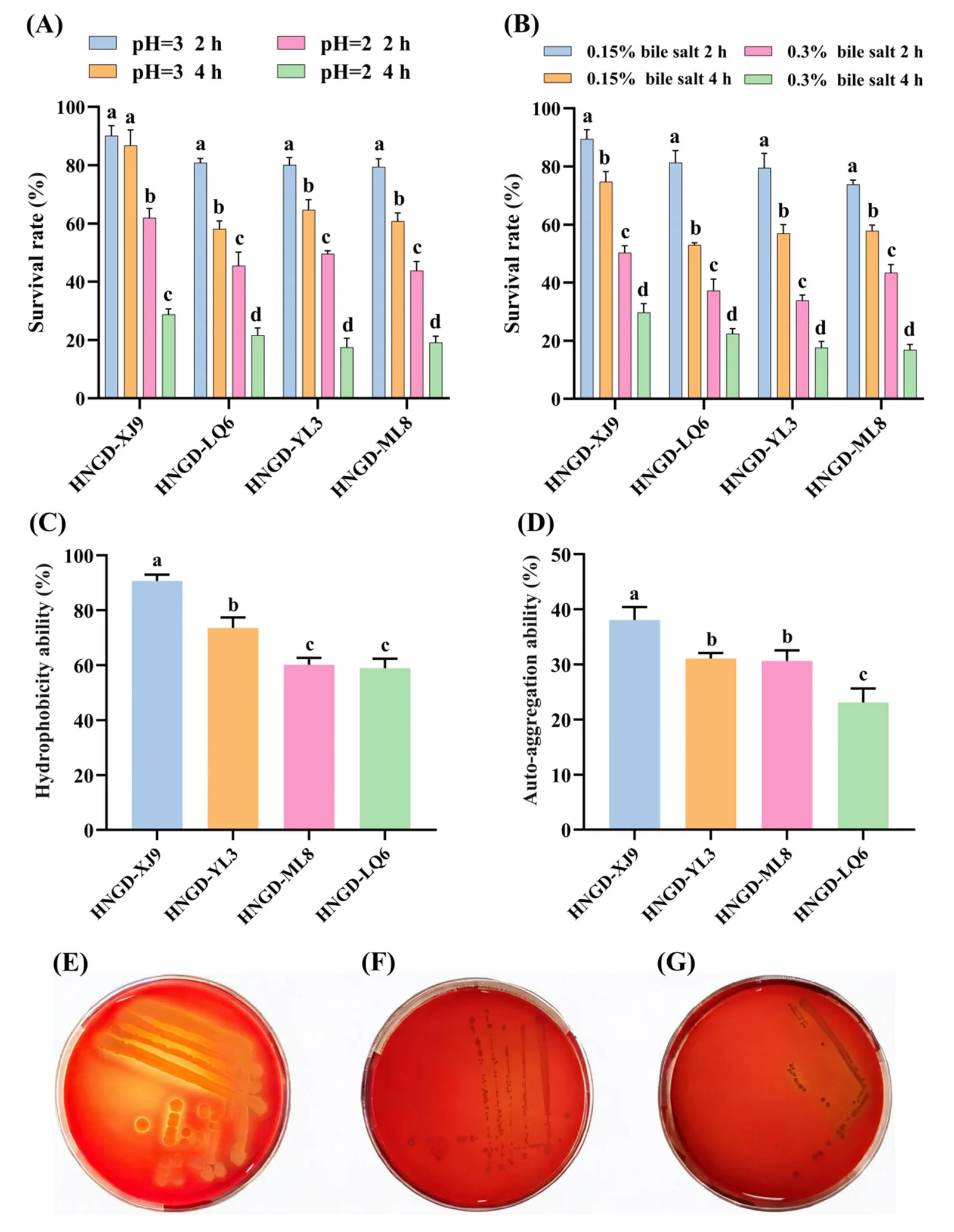
In vitro probiotic-associated characteristics and hemolytic activity of the candidate strains. (**A**) Acid tolerance (pH 2.0 and 3.0). (**B**) Bile salt tolerance (0.15% and 0.3%). (**C**) Cell surface hydrophobicity. (**D**) Auto-aggregation capacity. (**E**–**G**) Hemolytic activity: (**E**) β-hemolysis control, *Staphylococcus aureus* ATCC 25923; (**F**) γ-hemolysis control, *Lactiplantibacillus plantarum* ATCC 14917; (**G**) HNGD-XJ9 (γ-hemolysis). The red background corresponds to the blood agar medium; clear zones surrounding colonies are indicative of β-hemolysis. Values are presented as mean ± SEM from three independent biological replicates (*n* = 3). In panels (**A**–**D**), values with different lowercase letters (a, b, c, d) denote significant difference (*p* < 0.05), whereas identical letters indicate no significant difference.

Survival under the harsh gastrointestinal tract is a fundamental prerequisite for probiotic strains to confer health benefits on the host [43]. The acidic environment of the human stomach (pH 2–4) imposes strong selection pressure on ingested microorganisms, making acid tolerance a critical screening criterion for potential probiotics [44]. In this study, the survival rates of the four candidate strains under simulated gastric and intestinal conditions are presented in Figure 2A,B. After 2 h at pH 3.0, all four strains retained viability exceeding 75%, with HNGD-XJ9 showing the highest survival (approximately 90%). After 4 h at pH 3.0, survival rates were 86.77% (HNGD-XJ9), 58.16% (HNGD-LQ6), 64.70% (HNGD-YL3), and 60.76% (HNGD-ML8). However, under the harsher pH 2.0 condition, viability declined substantially, dropping to approximately 29% after 4 h for HNGD-XJ9, which was significantly greater than the other three strains (*p* < 0.05). The superior acid resistance of HNGD-XJ9 may be attributed to enhanced F_0_F_1_-ATPase function, as studies have confirmed that acid-resistant LAB exhibit elevated expression of this proton transporter under acidic stress [45]. Similar observations have been documented in previous reports, where extreme acidity, particularly at pH 2, was shown to substantially reduce LAB viability, with only strains possessing robust acid-adaptive mechanisms maintaining higher survival rates [46]. Compared with other *L. plantarum* strains, the acid tolerance of HNGD-XJ9 was moderate. For instance, *L. plantarum* ZZM01 exhibited 71.92% viability following 3 h at pH 2.0 [47], and *L. plantarum* OC-D showed 61.20% survival after 2 h in simulated gastric juice at pH 2.5 [48]. These conditions were less stringent than those used for HNGD-XJ9 (pH 2.0, 4 h). Although the approximately 29% survival of HNGD-XJ9 after 4 h at pH 2.0 was lower than that of ZZM01 and OC-D under their respective conditions, it indicates that HNGD-XJ9 possesses moderate acid tolerance.

Bile salt tolerance is another critical factor influencing probiotic survival and colonization within the gastrointestinal tract [49]. Following 2 h of exposure to 0.15% bile salts (Figure 2B), HNGD-XJ9 and HNGD-LQ6 maintained survival rates above 80%, while HNGD-YL3 (79.46%) and HNGD-ML8 (73.81%) were slightly lower. When the bile salt concentration was raised to 0.3%, the survival of HNGD-XJ9 dropped to approximately 50% at 2 h and 30% at 4 h; nevertheless, it still exhibited the highest survival among the four strains. The reduction in viability under bile salt stress is in line with the widely reported membrane-disrupting effects of bile salts, which compromise cell integrity and induce oxidative stress in sensitive strains [50]. The relatively better performance of HNGD-XJ9 under lower bile salt concentrations may be associated with the presence of bile resistance determinants, such as bile salt hydrolase (BSH), which has been reported to confer protective effects against bile-induced damage in *Lactobacillus* species through deconjugation of bile salts [51,52]. Under the same conditions (0.3% bile salts, 4 h), *L. plantarum* ZZM01 exhibited 84.71% survival [47] and *L. plantarum* OC-D showed 84.50% survival [48], whereas HNGD-XJ9 showed approximately 30% survival. The bile salt tolerance of HNGD-XJ9 was lower than that of these two strains under the same conditions, which may reflect strain-specific differences in bile resistance mechanisms, such as BSH activity.

#### 3.2.2. Cell Surface Hydrophobicity and Auto-Aggregation

Effective attachment to intestinal epithelial cells is regarded as a key determinant of potential probiotic persistence and functionality in the gut [53]. Among the various approaches for assessing this trait, auto-aggregation and cell surface hydrophobicity are frequently used as indicators of adhesive potential [54]. Figure 2C shows that HNGD-XJ9 displayed markedly higher hydrophobicity (90.66%) relative to HNGD-ML8, HNGD-LQ6, and HNGD-YL3 (*p* < 0.05). Strong hydrophobicity is thought to promote the initial interaction between bacterial cells and the intestinal mucosa, potentially facilitating adhesion [55]. Previous investigations have reported that *Lactobacillus* strains with pronounced hydrophobicity tend to exhibit enhanced mucosal binding, whereas those with low hydrophobicity show reduced intestinal retention and diminished probiotic-related effects [56]. The hydrophobicity of HNGD-XJ9 (90.66%) was higher than the values reported for *L. plantarum* ZZM01 (33.19%) [47] and OC-D (58.56%) [48].

A comparable trend emerged for auto-aggregation (Figure 2D), with HNGD-XJ9 again outperformed the other three strains, achieving the highest value (38.04%) (*p* < 0.05). The capacity to auto-aggregate is considered advantageous for probiotic bacteria, as it supports adhesion to epithelial cells and competitive exclusion of pathogens [57]. The auto-aggregation observed in HNGD-XJ9 may reflect the presence of surface-associated components, including S-layer proteins or exopolysaccharides; prior work have demonstrated that removing S-layer from *Lactobacillus* cells can reduce auto-aggregation by 11–90% [58]. Such aggregation properties have been associated with improved gut retention and anti-pathogenic activity, as documented for various *lactobacilli* that rely on surface proteins to interfere with pathogen attachment [59]. The auto-aggregation of HNGD-XJ9 (38.04%) was comparable to that of *L. plantarum* OC-D (40.38%) [48], but lower than that of ZZM01 (93.85% at 24 h) [47]. These differences may reflect strain-specific surface properties and assay conditions.

Based on the integrated assessment of acid/bile salt tolerance, auto-aggregation, and hydrophobicity, HNGD-XJ9 exhibited the most favorable overall profile and was consequently chosen for subsequent characterization and application studies.

#### 3.2.3. Hemolytic Activity

Hemolysis by strain HNGD-XJ9 was assessed on Columbia blood agar plates as part of the safety assessment. As shown in Figure 2E–G, the positive reference strain *Staphylococcus aureus* ATCC 25923 produced clear rings around its colonies, indicating β-hemolytic activity (Figure 2E). By contrast, no such zones were produced by either the negative reference strain *Lactiplantibacillus plantarum* ATCC 14917 or HNGD-XJ9, consistent with γ-hemolysis (Figure 2F,G). These observations indicate that HNGD-XJ9 showed no hemolytic activity under the conditions tested. This result is consistent with previous reports on other *L. plantarum* strains [60]. Similarly, *L. plantarum* ZZM01 and OC-D also showed no hemolytic activity [47,48]. Further tests are required to fully evaluate its safety.

### 3.3. Identification, Morphological, and Physiological Characterization of HNGD-XJ9

**Figure 3:**
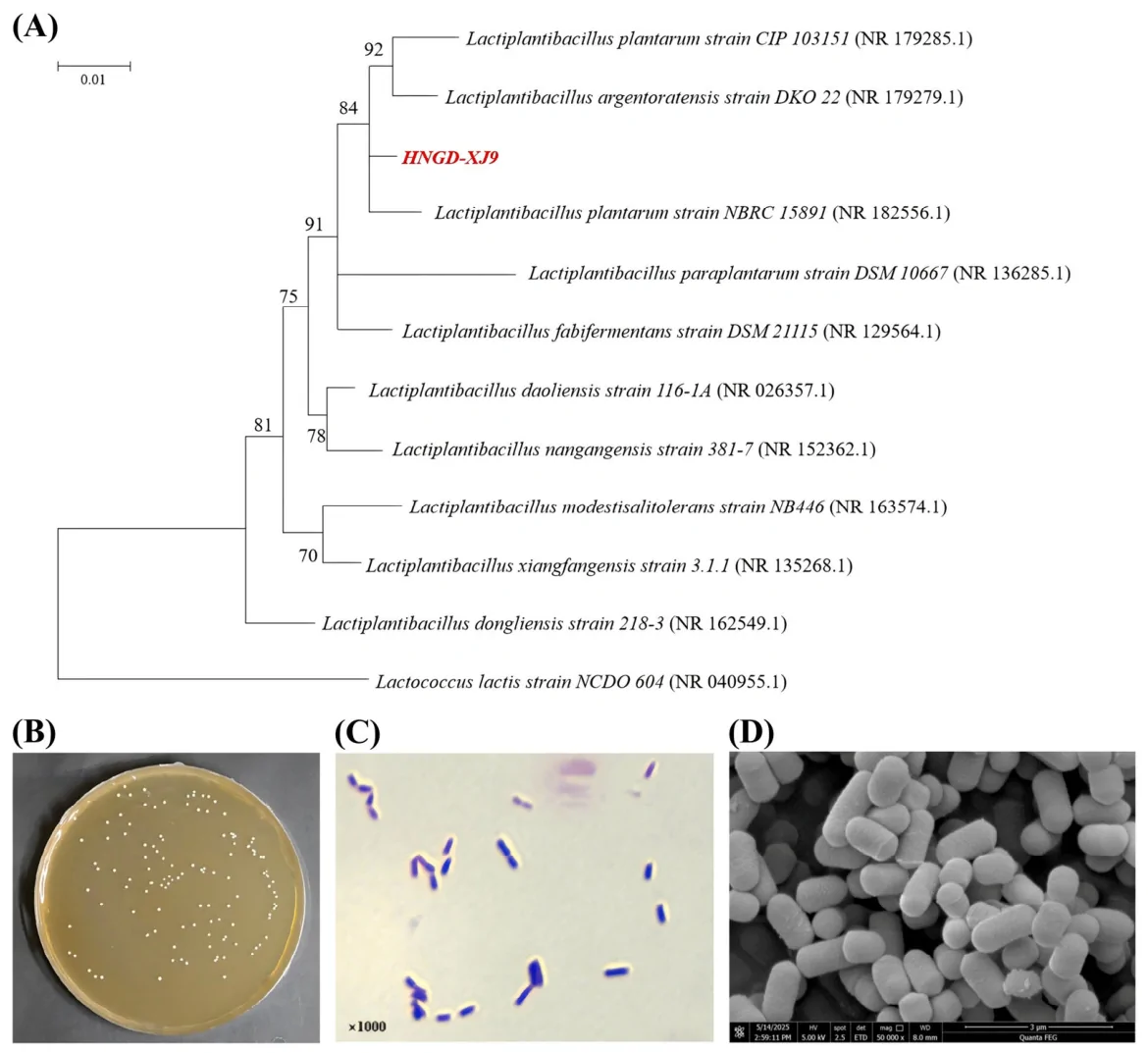
Taxonomic assignment and morphological features of HNGD-XJ9. (**A**) Phylogenetic tree. (**B**) Colony morphology on MRS agar. (**C**) Gram staining (×1000). (**D**) Scanning electron microscopy (SEM) image (×50,000).

The taxonomic affiliation of HNGD-XJ9 was determined by whole-genome ANI analysis, 16S rDNA gene sequencing, and phylogenetic analysis. As shown in Figure 3A, the strain clustered within a clade containing multiple *Lactiplantibacillus plantarum* strains with a bootstrap value of 92%, indicating its classification as *L. plantarum*. Species identity was further verified by ANI analysis between the type strain *L. plantarum* ATCC 14917^T^ and HNGD-XJ9. The resulting ANI value of 99.18% exceeded the 95% species cutoff, confirming HNGD-XJ9 as *L. plantarum*. On MRS agar, circular, convex, milky white colonies with regular contours and glossy surfaces were observed (Figure 3B). Gram staining showed the characteristic purple coloration of Gram-positive cells (Figure 3C), while scanning electron microscopy further revealed the typical rod-shaped bacillary form (Figure 3D). These morphological features align with the established description of *L. plantarum* [61,62].

**Figure 4:**
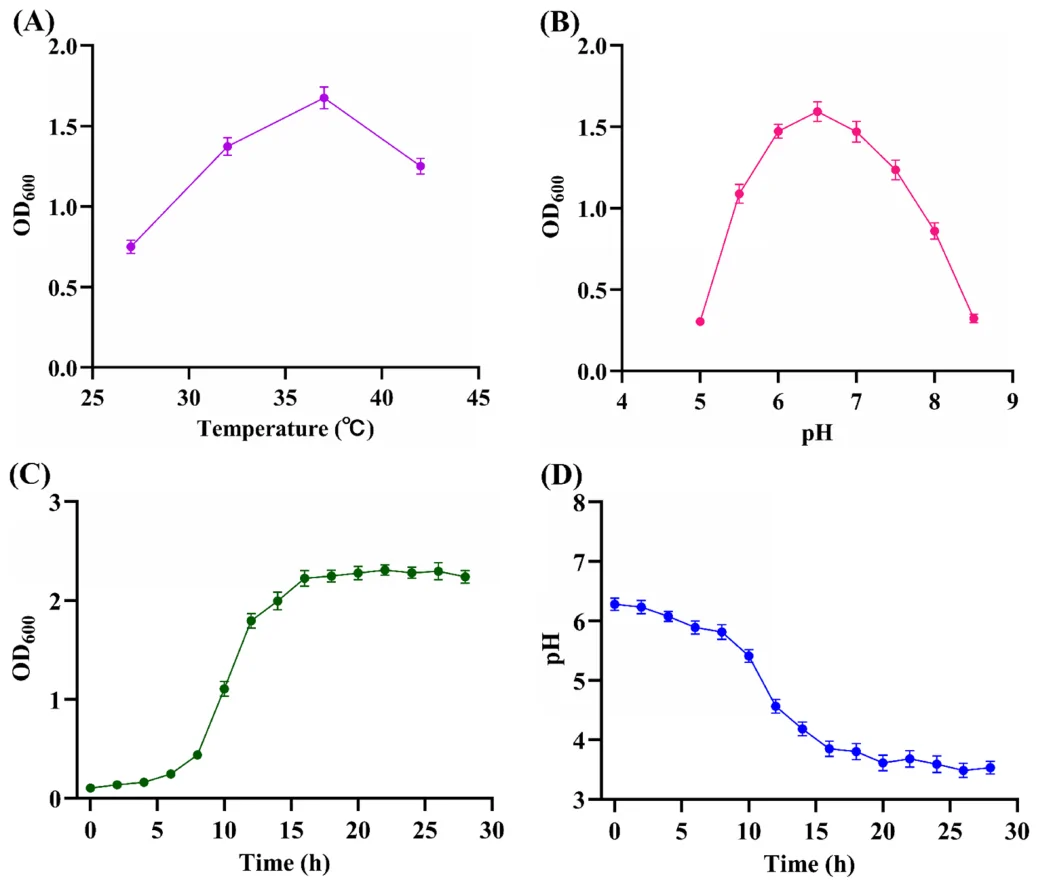
Growth characteristics of strain HNGD-XJ9. (**A**) Growth at different temperatures after 24 h of cultivation. (**B**) Growth under various initial pH conditions (24 h at 37 °C). (**C**) Growth curve and (**D**) pH changes during fermentation. Values are presented as mean ± SEM from three independent biological replicates (*n* = 3).

The growth behavior of HNGD-XJ9 was investigated by examining the influence of temperature and initial pH on its proliferation. The highest biomass accumulation was recorded at 37 °C, while initial pH 6.5 yielded maximal growth (Figure 4A,B). These optimal parameters are consistent with previously reported growth conditions for *L. plantarum* strains [63]. Employing these optimized parameters, the growth curve and pH changes of HNGD-XJ9 were monitored over time (Figure 4C,D). The lag phase extended from 0 to 8 h, during which the OD_600_ rose gradually from 0 to 0.44. The culture then entered exponential growth between 8 and 12 h, with OD_600_ increasing rapidly to 1.79. The stationary phase was maintained from 12 to 28 h, with the maximum OD_600_ reaching 2.31. In parallel, the medium pH fell from an initial value of 6.28 to 3.85 during the exponential and subsequent early stationary phases (8–16 h), indicating strong acid-producing capacity. This inverse correlation between cell growth and pH decline is characteristic of LAB fermentation and has been documented in various *Lactobacillus* species [22,64]. The acidification profile of HNGD-XJ9 suggests its potential utility in food fermentation applications, where rapid pH reduction is a key factor for ensuring microbial safety and product stability [65].

### 3.4. Whole-Genome Sequencing and Genomic Analysis

#### 3.4.1. Genome Assembly and Functional Annotation

**Figure 5:**
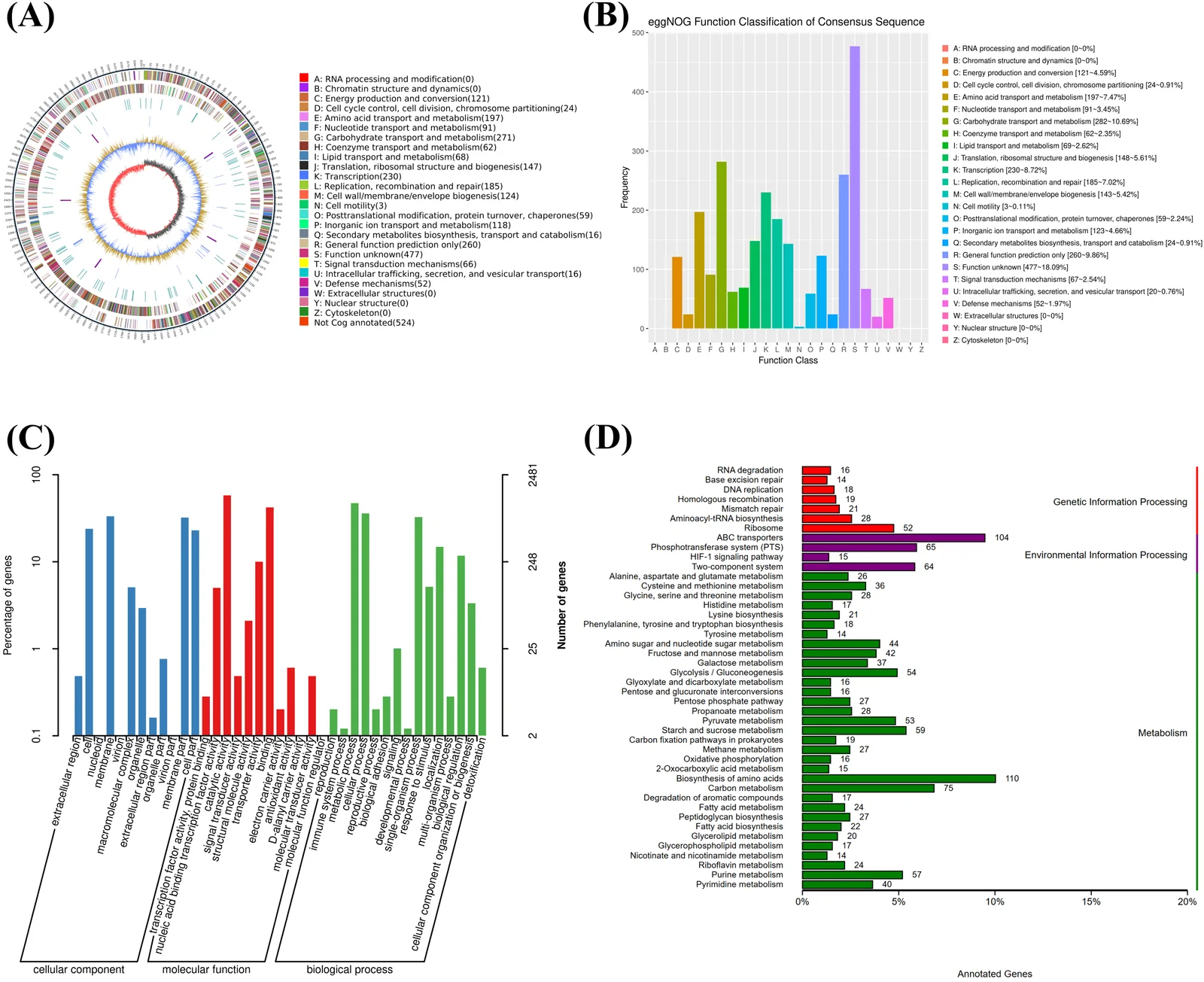
Genomic features and functional annotation of HNGD-XJ9. (**A**) Circular representation of the HNGD-XJ9 genome. (**B**) eggNOG functional categories. (**C**) GO functional categories (blue: cellular component; red: molecular function; green: biological process). (**D**) KEGG pathway classification (red: genetic information processing; purple: environmental information processing; green: metabolism).

To elucidate the genetic basis underlying the dityrosine-lowering capability of HNGD-XJ9, whole-genome sequencing and comprehensive functional annotation were carried out. The genome comprises one circular chromosome (3,355,384 bp; 44.42% GC). Annotation of the genome predicted 4,240 protein-coding sequences, alongside 75 tRNA and 16 rRNA genes (Figure 5A). These genomic features are similar to those typically found in *L. plantarum* strains [66,67]. Functional annotation was performed using Evolutionary Genealogy of Genes: Non-supervised Orthologous Groups (eggNOG), Gene Ontology (GO), KEGG, Carbohydrate-active enzymes (CAZymes), and other public databases. eggNOG annotation assigned 2587 genes (Figure 5B), with pronounced enrichment in carbohydrate transport and metabolism (271 genes), amino acid transport and metabolism (197 genes), and energy production and conversion (121 genes). The enrichment in amino acid transport and metabolism is particularly relevant, given that dityrosine is a dimeric derivative of tyrosine. GO analysis categorized 2,481 genes (Figure 5C), with a notable abundance of genes involved in catalytic activity and metabolic processes within the molecular function category. KEGG pathway analysis (Figure 5D) identified notable enrichment in pathways directly relevant to dityrosine reduction, including tyrosine metabolism (14 genes) and degradation of aromatic compounds (17 genes). Additionally, biosynthesis of amino acids (110 genes), carbon metabolism (75 genes), and ABC transporters (104 genes) were among the most abundant categories, supporting the metabolic versatility and substrate transport capacity of HNGD-XJ9.

#### 3.4.2. CAZyme Analysis

**Figure 6:**
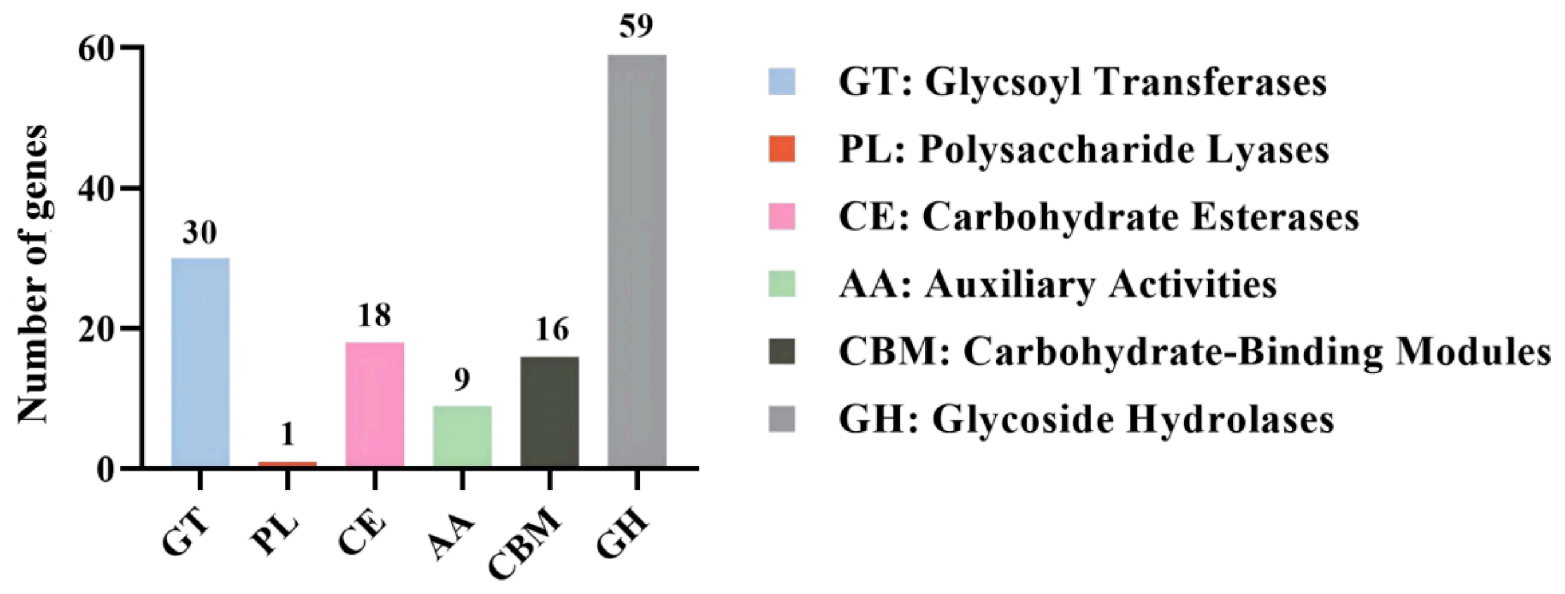
Distribution of CAZyme family genes in the genome of strain HNGD-XJ9. Numbers above bar indicate the number of genes in each CAZyme family.

CAZyme annotation of the HNGD-XJ9 genome identified a total of 133 carbohydrate-active enzyme genes (Figure 6). Glycosyl transferases (GT, 30 genes) and glycoside hydrolases (GH, 59 genes) were the predominant CAZyme families; the remaining families included polysaccharide lyases (PL, 1 gene), auxiliary activities (AA, 9 genes), carbohydrate-binding modules (CBM, 16 genes), and carbohydrate esterases (CE, 18 genes). GH enzymes are primarily responsible for hydrolyzing glycosidic bonds in complex carbohydrates, whereas GTs participate in the biosynthesis of polysaccharides and oligosaccharides [68]. The abundance of GT and GH genes indicates that HNGD-XJ9 has a robust enzymatic system for carbohydrate metabolism, which may underpin its adaptability to diverse carbohydrate substrates in fermented food environments [69].

#### 3.4.3. Identification of Candidate Genes Potentially Involved in Dityrosine Reduction

To identify candidate genes potentially involved in dityrosine reduction, a targeted screening of the annotated genome was performed, focusing on genes associated with aromatic amino acid metabolism and aromatic ring cleavage. This approach identified three candidate genes (Table S1). GE001281 encodes an enzyme associated with aromatic amino acid metabolism (eggNOG category [E]), which may participate in the catabolism of the tyrosine backbone and potentially contribute to the disruption of C–C crosslinks between tyrosine residues [70,71]. GE001328 encodes a homogentisate dioxygenase that can catalyze oxidative ring cleavage of aromatic structures [72]. GE002892 encodes an aromatic ring dioxygenase that may specifically catalyze the oxidative cleavage of aromatic conjugated systems [73,74]. The identification of genes encoding homogentisate dioxygenase (GE001328) and aromatic ring dioxygenase (GE002892) is particularly noteworthy, as these enzymes are known to catalyze the oxidative cleavage of aromatic ring structures [73]. Given that dityrosine is a biphenyl-type dimer formed by C–C crosslinking between two tyrosine residues [1], its putative degradation likely requires two sequential steps: cleavage of the inter-tyrosine C–C bond, followed by oxidative ring opening of the tyrosine aromatic rings. The enzymes encoded by GE001328 and GE002892 may participate in the latter step, while GE001281, involved in aromatic amino acid metabolism, may contribute to the former. This proposed two-step model for dityrosine reduction by HNGD-XJ9 is analogous to the multi-enzymatic degradation pathways reported for other recalcitrant aromatic compounds [73,75]; however, it remains a hypothesis and requires experimental validation. The enrichment of tyrosine metabolism and aromatic compound degradation pathways in the KEGG annotation further supports this proposed mechanism.

#### 3.4.4. In Silico Assessment of Potential Safety-Related Genes

In silico screening of the HNGD-XJ9 genome was performed to identify potential antibiotic resistance and virulence determinants, using the criteria described in Section 2.6. Four antibiotic resistance-associated sequences were identified, with identities to known resistance genes ranging from 27.41% to 45.12% (Table S2). These sequences were assigned as Loose hits, and no mobile genetic elements (e.g., plasmids, transposons, or integrons) were found adjacent to them, indicating a low likelihood of horizontal gene transfer [60]. In addition, a BLAST search against Pathogen–Host Interaction database (PHI-base) revealed only four sequences showing low similarity to known pathogenicity-related sequences, with identity values of 26.42–41.25% (Table S3). Based on these in silico criteria, the HNGD-XJ9 genome did not contain any complete or high-confidence acquired antibiotic resistance genes or virulence determinants. These genomic findings suggest a low potential safety risk and support further investigation of HNGD-XJ9 as a potential food-grade candidate strain. However, it should be emphasized that sequence-based predictions cannot definitively exclude the presence of functional resistance or virulence factors, and phenotypic antibiotic susceptibility testing and virulence assays are required for a comprehensive safety assessment [34].

### 3.5. Impact of HNGD-XJ9 on Protein Oxidation in Spray-Dried Milk Powder

**Figure 7:**
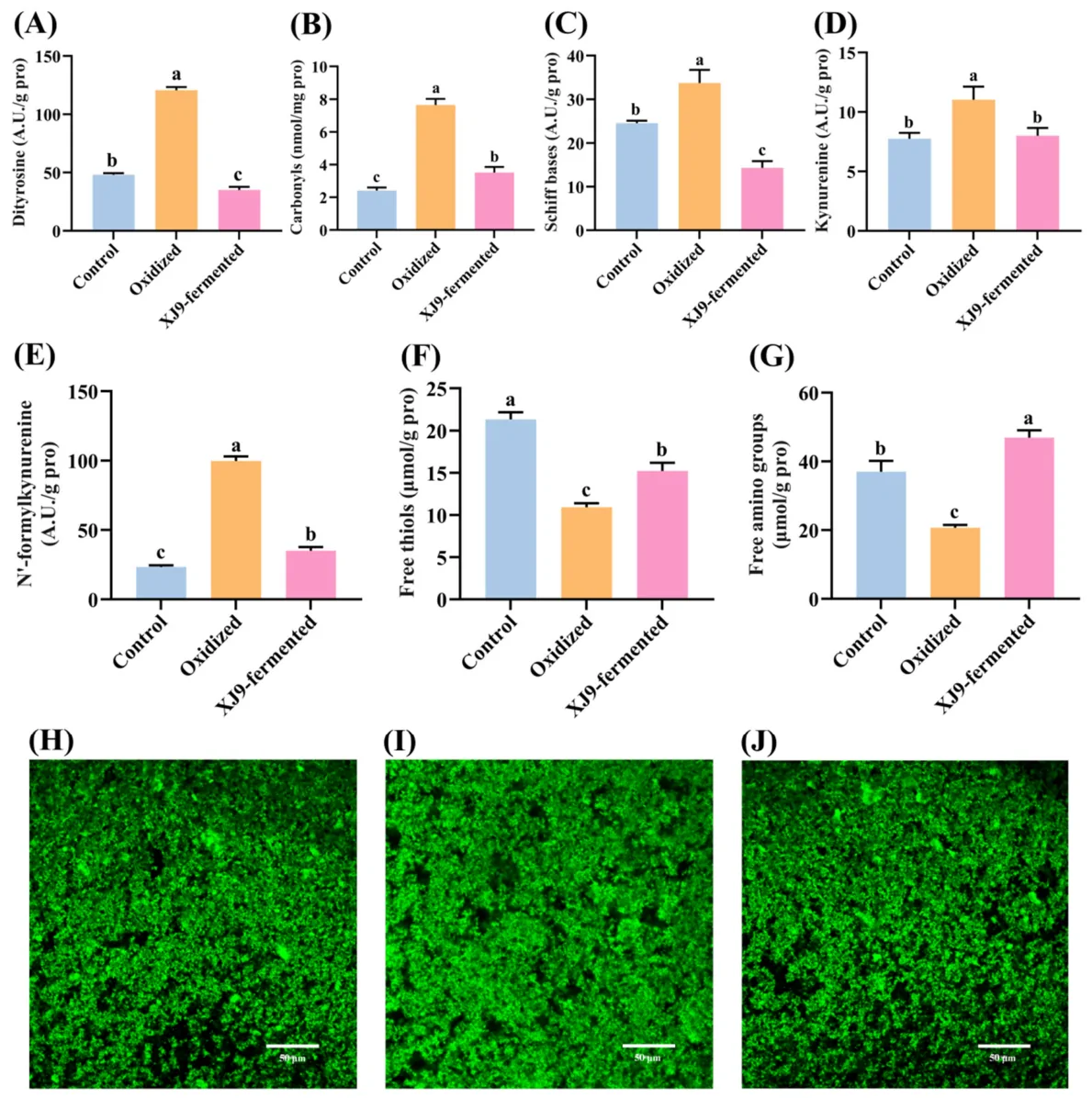
Impact of HNGD-XJ9 fermentation on oxidative modifications of spray-dried milk powder proteins. (**A**–**G**) Contents of dityrosine, carbonyls, Schiff bases, kynurenine, N′-formylkynurenine, free thiols, and free amino groups. Confocal laser scanning microscopy (CLSM) micrographs of milk proteins samples: (**H**) control, (**I**) oxidized, and (**J**) XJ9-fermented samples. Scale bar = 50 μm. Values are presented as mean ± SEM from three independent biological replicates (*n* = 3). For panels (**A**–**G**), values with different lowercase letters (a, b, c) denote significant difference (*p* < 0.05), whereas identical letter indicates no significant difference.

To evaluate whether HNGD-XJ9 could reduce protein oxidation products in a real food system, spray-dried milk powder was selected as the first model. Freeze-dried pasteurized milk powder was designated as the low-oxidation reference, whereas its spray-dried counterpart constituted the high-oxidation sample. The data presented in Figure 7A–G revealed that the oxidized milk powder contained markedly higher levels of dityrosine, carbonyls, kynurenine, N′-formylkynurenine and Schiff bases than the control (*p* < 0.05), alongside a significant depletion of free amino groups and free thiols (*p* < 0.05). These changes confirm that spray-drying caused severe oxidative damage to milk proteins, consistent with previous observations that thermal processing induces protein oxidation [1,42]. After 48 h of fermentation with HNGD-XJ9, the dityrosine level in the oxidized milk powder was reduced by 70.89% (Figure 7A), and the other four oxidation markers (carbonyls, kynurenine, N′-formylkynurenine, and Schiff bases) were also significantly reduced (*p* < 0.05). Simultaneously, free amino groups and free thiols were markedly restored to levels approaching those of the control (*p* < 0.05). These findings indicate that HNGD-XJ9 fermentation substantially attenuates multiple oxidative modifications in milk powder, a finding that is consistent with the putative enzymatic mechanisms underlying dityrosine reduction inferred from genomic analysis.

To gain further insight into the structural impact of HNGD-XJ9 fermentation, CLSM was employed to visualize protein morphology (Figure 7H–J). The control sample displayed uniformly dispersed small particles with stable fluorescence, suggesting well-preserved protein structure (Figure 7H). In contrast, the oxidized milk powder exhibited pronounced aggregation, with bright fluorescent patches corresponding to crosslinked protein clusters, a hallmark of oxidation-induced protein crosslinking [76] (Figure 7I). Notably, the increased fluorescence intensity within these aggregates may be attributed to the higher local protein concentration, which provides more binding sites for FITC despite the possible masking of some amino groups by crosslinks. After fermentation with HNGD-XJ9, the aggregated structures largely disappeared, and the protein particles became dispersed again, implying effective disruption of the crosslinked network (Figure 7J).

FTIR spectroscopy was further applied to assess the secondary structure features of milk proteins (Table 1). The control sample displayed 22.04% α-helix and 33.47% β-sheet, characteristic of a well-preserved ordered structure. Following spray-drying, the α-helix content slightly decreased to 20.13%, whereas the β-sheet content increased markedly to 42.87% (*p* < 0.05), accompanied by a significant reduction in β-turn from 28.16% to 17.38% (*p* < 0.05). Random coil content also rose modestly, from 16.33% to 19.62%. This conformational shift indicates that spray-drying disrupted the secondary structure equilibrium, driving the proteins toward more aggregated states, consistent with previous FTIR studies on thermally processed milk proteins [42]. Similar structural alterations have also been observed in other thermally processed food systems [77]. After fermentation with HNGD-XJ9, the α-helix content recovered to 21.52%, the β-sheet content decreased to 36.18% (*p* < 0.05), and β-turn content rose to 23.77% (*p* < 0.05). Overall, the secondary structure profile of the fermented sample approached that of the unoxidized control, indicating that HNGD-XJ9 fermentation reduced oxidation products while also partially recovers the native protein conformation. This structural recovery might be facilitated by the enzymatic activities of the strain, including proteases and esterases that potentially hydrolyze crosslinked peptide bonds and ester linkages; however, this hypothesis is based solely on genomic predictions of candidate genes and requires direct enzymatic validation. Similar structural recovery of proteins by LAB fermentation has been reported in other food systems [78].

### 3.6. Impact of HNGD-XJ9 on Protein Oxidation in Roasted Pork Powder

**Figure 8:**
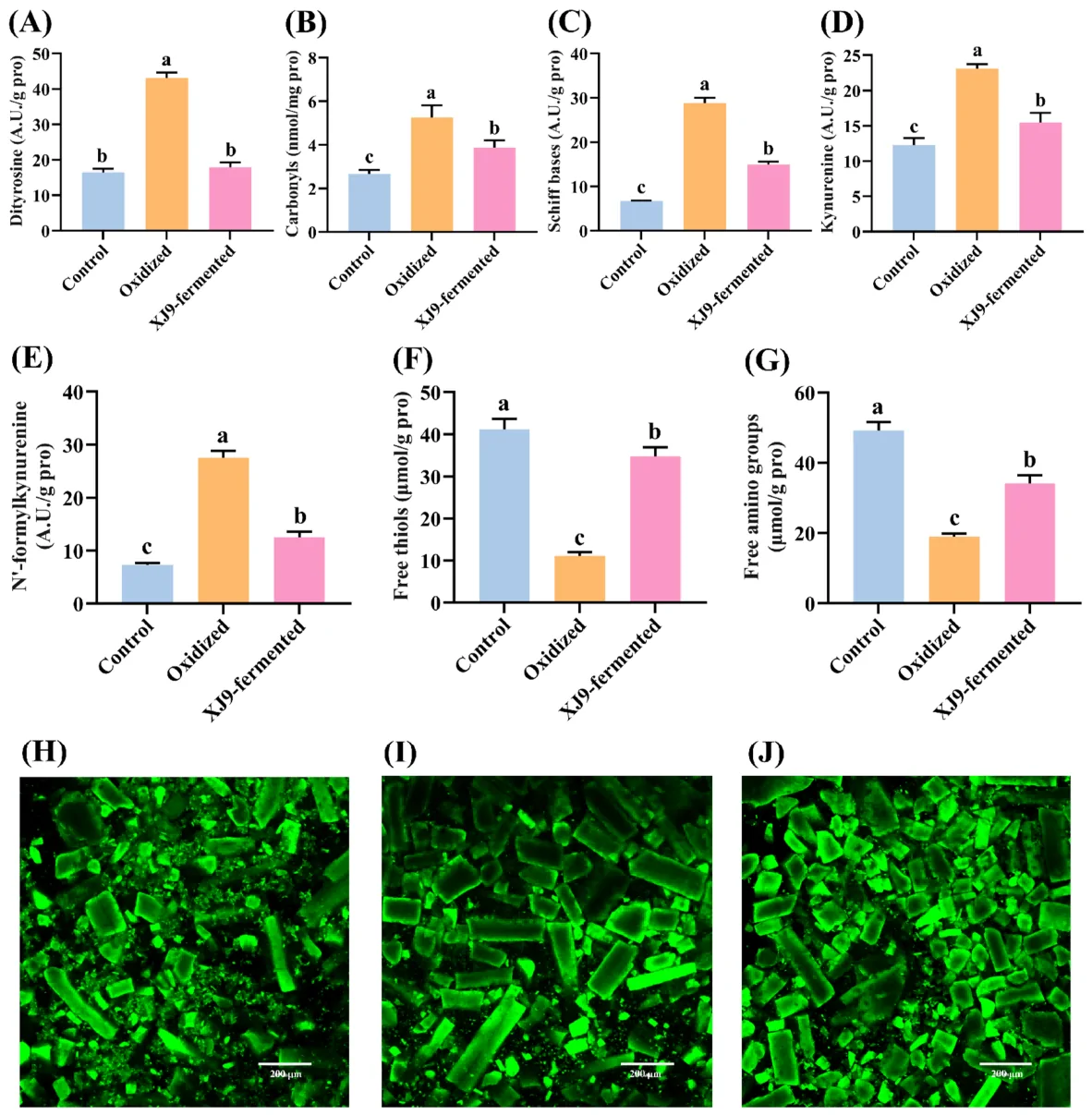
Impact of HNGD-XJ9 fermentation on oxidative modifications of roasted pork powder proteins. (**A**–**G**) Contents of dityrosine, carbonyls, Schiff bases, kynurenine, N′-formylkynurenine, free thiols, and free amino groups. CLSM micrographs of pork protein samples: (**H**) control, (**I**) oxidized, and (**J**) XJ9-fermented samples. Scale bar = 200 μm. Values are presented as mean ± SEM from three independent biological replicates (*n* = 3). For panels (**A**–**G**), values with different lowercase letters (a, b, c) denote significant difference (*p* < 0.05), whereas identical letter indicates no significant difference.

To further confirm the capacity of HNGD-XJ9 to reduce protein oxidation products in an alternative food matrix, roasted pork powder was selected as the second model. Pork powder prepared by sous-vide cooking (65 °C, 10 h) served as the low-oxidation reference, whereas the high-oxidation counterpart was obtained by oven roasting (180 °C, 30 min). Data presented in Figure 8A–G revealed that roasting led to a marked elevation in dityrosine, carbonyls, kynurenine, N′-formylkynurenine, and Schiff bases levels compared with the control (*p* < 0.05), while causing a significant depletion of free amino groups and free thiols (*p* < 0.05). These results confirm that roasting at high-temperature induced pronounced protein oxidation in pork powder, consistent with the well-established oxidative effects of thermal processing on meat proteins [1,79]. Notably, the fold-increase in dityrosine levels in roasted pork powder was comparable to that in milk powder, indicating that both thermal processing methods caused substantial protein oxidation despite the different protein matrices. After 48 h of fermentation with HNGD-XJ9, the dityrosine level in the oxidized sample was diminished by 58.61% (Figure 8A), a reduction slightly lower than the 70.89% observed in the milk powder system. The other four oxidative markers (carbonyls, N′-formylkynurenine, kynurenine, and Schiff bases) also decreased markedly (*p* < 0.05), while free amino groups and free thiols were restored to levels markedly higher than in the unfermented oxidized sample (*p* < 0.05). These results are in line with earlier reports that fermentation with *L. plantarum* or other LAB can reduce protein oxidation in meat products, such as decreased surface hydrophobicity and carbonyl content in fermented sausage [20] and air-dried beef [80]. However, to our knowledge, this is the first report of dityrosine reduction in a meat matrix by LAB fermentation. Myofibrillar proteins, which constitute the major protein component of meat, possess a highly organized and compact fibrous structure [81]. The inherent structural rigidity of this protein matrix may limit bacterial accessibility and enzymatic action, partly accounting for the slightly lower reduction rates observed in the meat system compared to the milk powder system.

To further investigate the structural impact of HNGD-XJ9 fermentation on pork proteins, CLSM was employed to visualize protein morphology (Figure 8H–J). The control sample showed a uniform dispersion of small particles with stable fluorescence, suggesting the structural integrity of myofibrillar proteins (Figure 8H). Conversely, in the roasted pork powder, numerous elongated and densely packed myofibrillar assemblies with well-defined contours and intensified fluorescence were evident, reflecting thermally induced crosslinking that increased the structural rigidity of the proteins (Figure 8I). This observation aligns with the known susceptibility of muscle proteins to oxidative crosslinking during thermal processing, which promotes aggregate formation and reduces protein extractability [81]. Notably, the overall fluorescence intensity in the oxidized group did not increase proportionally with structural densification, likely because crosslinking partially masked free amino groups, thereby reducing the number of available FITC binding sites. Following fermentation, these elongated myofibrillar structures were largely disrupted, and the proteins were converted into thicker, shorter, and more uniformly shaped particles with markedly enhanced fluorescence, indicating that HNGD-XJ9 fermentation effectively mitigated protein aggregation and crosslinking in the meat matrix (Figure 8J). These results agree with earlier studies demonstrating that fermentation with LAB can directly hydrolyze myofibrillar proteins [82] and induce structural changes that may underlie the partial structural recovery observed in this study.

FTIR spectroscopy was employed to monitor the conformational changes in pork proteins (Table 2). The control sample displayed α-helix and β-sheet fractions of 24.52% and 34.08%, respectively, characteristic of a well-preserved native protein structure. Roasting at high temperature induced a pronounced decrease in α-helix to 12.28% (*p* < 0.05) and a concomitant increase in β-sheet content to 47.53% (*p* < 0.05), along with a significant rise in random coil from 10.47% to 19.92% (*p* < 0.05) and a reduction in β-turn from 30.93% to 20.27% (*p* < 0.05). This conformational shift, characterized by the loss of ordered α-helical structures and the accumulation of β-sheet and random coil conformations, reflects severe disruption of the secondary structure equilibrium induced by thermal oxidation. Notably, the magnitude of structural change in pork proteins was greater than that observed in milk proteins, consistent with the more pronounced oxidation levels in the meat matrix. After fermentation with HNGD-XJ9, the α-helix content recovered to 16.57% (*p* < 0.05), the β-sheet content decreased to 37.42% (*p* < 0.05), and β-turn increased to 32.91% (*p* < 0.05), approaching the control levels. The observed reduction in β-sheet and the partial restoration of α-helix following fermentation differ from the trends commonly reported for LAB-fermented meat products, where α-helix typically decreases and β-sheet increases [83,84]. However, this trend is consistent with findings in other protein systems; for instance, fermentation with *L. pentosus* significantly lowered β-sheet and raised α-helix of peanut protein [85]. These discrepancies suggest that the effect of LAB fermentation on protein secondary structure is strain- and matrix-dependent. In the present study, HNGD-XJ9 fermentation partially recovers the native protein conformation in oxidized pork powder, with the extent of structural recovery corresponding to that observed in the milk powder system. This structural recovery may be partly associated with the enzymatic activities of HNGD-XJ9, particularly those of proteases and esterases, which could disrupt crosslinked peptide and ester bonds within the oxidized meat protein matrix. This hypothesis is based on the genomic identification of candidate genes potentially involved in such hydrolytic activities; however, no direct enzymatic evidence was obtained in this study, and further validation is required.

Several limitations of this study should be acknowledged. The fluorescence assay cannot fully distinguish true dityrosine degradation from adsorption, precipitation, dilution, or other physicochemical changes, owing to the lack of cell-free supernatant and heat-killed cell controls, and dityrosine quantification was not validated with an authentic standard, calibration curve, recovery test, or LC-MS/MS or HPLC. The roles of the candidate genes (GE001281, GE001328, and GE002892) were deduced only from genome annotation and have not been functionally validated; these genes should therefore be regarded as candidates and the proposed pathway remains putative. In addition, the probiotic efficacy and safety of HNGD-XJ9 were not fully established, as acid/bile tolerance, hydrophobicity, auto-aggregation, and hemolysis are only in vitro indicators, and antibiotic susceptibility was not assessed phenotypically. The structural recovery observed after fermentation is hypothesized to be associated with enzymatic activities, but no direct enzymatic evidence was obtained. Future work combining LC-MS/MS quantification of dityrosine, validation of gene function, suitable fermentation controls, phenotypic antibiotic susceptibility assays, and in vivo experiments are required to verify the mechanism, safety, and potential utilizations of HNGD-XJ9.

## 4. Conclusions

In the present work, *Lactiplantibacillus plantarum* HNGD-XJ9 was screened from a collection of naturally fermented food isolates for its high dityrosine-lowering activity. The strain exhibited favorable gastrointestinal tolerance, notable adhesive properties, and no hemolytic activity under the tested conditions, supporting its further investigation as a potential food-grade candidate. Genomic profiling identified candidate genes (GE001281, GE001328, and GE002892) associated with aromatic amino acid metabolism and aromatic ring cleavage, providing putative molecular-level evidence for its dityrosine-lowering capacity. When applied to thermally processed foods, HNGD-XJ9 fermentation reduced detectable dityrosine levels by 70.89% and 58.61% in spray-dried milk powder and roasted pork powder, respectively, simultaneously mitigating other oxidative modifications and partially recovering protein conformational integrity. These findings suggest that HNGD-XJ9 could be applied in the food industry to reduce pre-existing dityrosine and other protein oxidation products in thermally processed dairy and meat products. This may contribute to the development of functional foods with reduced protein oxidation-related risks. Further experimental validation of the candidate genes is warranted to confirm the specific mechanisms.

## Figures and Tables

**Table 1:** Effects of HNGD-XJ9 fermentation on the secondary structure of milk powder proteins.

	α-Helix (%)	β-Sheet (%)	β-Turn (%)	Random Coil (%)
Control	22.04 ± 1.82 ^a^	33.47 ± 3.27 ^c^	28.16 ± 1.54 ^a^	16.33 ± 1.36 ^a^
Oxidized	20.13 ± 2.21 ^a^	42.87 ± 1.89 ^a^	17.38 ± 2.17 ^c^	19.62 ± 2.75 ^a^
XJ9-fermented	21.52 ± 1.15 ^a^	36.18 ± 2.25 ^b^	23.77 ± 2.76 ^b^	18.53 ± 1.47 ^a^

Note: Control: pasteurized milk powder (low-oxidation); Oxidized: spray-dried milk powder; XJ9-fermented: spray-dried milk powder fermented with HNGD-XJ9. Values are presented as mean ± SEM from three independent biological replicates (*n* = 3). For each column, values with different lowercase letters (a, b, c) denote significant difference (*p* < 0.05), whereas identical letter indicates no significant difference.

**Table 2:** Effects of HNGD-XJ9 fermentation on the secondary structure of pork powder proteins.

	α-Helix (%)	β-Sheet (%)	β-Turn (%)	Random Coil (%)
Control	24.52 ± 2.85 ^a^	34.08 ± 3.15 ^b^	30.93 ± 3.34 ^a^	10.47 ± 1.53 ^c^
Oxidized	12.28 ± 1.47 ^c^	47.53 ± 3.91 ^a^	20.27 ± 2.17 ^b^	19.92 ± 2.71 ^a^
XJ9-fermented	16.57 ± 1.16 ^b^	37.42 ± 2.52 ^b^	32.91 ± 3.83 ^a^	13.09 ± 1.26 ^b^

Note: Control: low-oxidation pork powder prepared by sous-vide cooking; Oxidized: oven-roasted pork powder; XJ9-fermented: oven-roasted pork powder fermented with HNGD-XJ9. Values are presented as mean ± SEM from three independent biological replicates (*n* = 3). For each column, values with different lowercase letters (a, b, c) denote significant difference (*p* < 0.05), whereas identical letter indicates no significant difference.

## Data Availability

The original contributions presented in this study are included in the article/Supplementary Materials. Further inquiries can be directed to the corresponding author.

## Supplementary Materials

The following supporting information can be downloaded at https://www.mdpi.com/article/10.3390/foods15213778/s1: Table S1: Candidate genes potentially related to dityrosine reduction in HNGD-XJ9; Table S2: Antibiotic resistance genes detected in the HNGD-XJ9 genome; Table S3: Potential pathogenicity-related genes in HNGD-XJ9 based on PHI-base annotation.

## Abbreviations

The following abbreviations are used in this manuscript:

LAB	Lactic acid bacteria
KEGG	Kyoto Encyclopedia of Genes and Genomes
eggNOG	Evolutionary Genealogy of Genes: Non-supervised Orthologous Groups
GO	Gene Ontology
CAZymes	Carbohydrate-active enzymes
PHI-base	Pathogen–Host Interaction database
CLSM	Confocal laser scanning microscopy
FITC	Fluorescein isothiocyanate
FTIR	Fourier-transform infrared
GH	Glycoside hydrolases
GT	Glycosyl transferases
CE	Carbohydrate esterases
CBM	Carbohydrate-binding modules
AA	Auxiliary activities
PL	Polysaccharide lyases
