# Supplementary material for: Dityrosine-Lowering Lactiplantibacillus plantarum HNGD-XJ9: Screening, Characterization, and Evaluation
Source: Foods. 2026 Oct 22;15(21):3778. doi: 10.3390/foods15213778 (PMC13763267; doi:10.3390/foods15213778)
Supplement: Supplementary file 1 [file foods-15-03778-s001.zip › foods-4567908-supplementary.pdf]

Supplementary Materials

Dityrosine-Lowering *Lactiplantibacillus plantarum* HNGD-XJ9: Screening, Characterization, and Evaluation

Yueting Ge[1], Kaile Chen[2], Zhenguo Tian[2], Hao Zheng[2], Zihao Jin[2], Chengjia Gu[1], Guowei Le[3], Yuhui Yang[2*]

[1] Henan Key Laboratory of Tea Plant Biology, College of Tea and Food Science, Xinyang Normal University, Xinyang 464000, China

[2] College of Food Science and Engineering, Henan University of Technology, Zhengzhou 450001, China

[3] State Key Laboratory of Food Science and Technology, School of Food Science and Technology, Jiangnan University, Wuxi 214122, China

* Correspondence: Yuhui Yang, E-mail: yangyuhui1992@126.com

College of Food Science and Engineering, Henan University of Technology

100 Lotus Street, Hi-tech Development District, Zhengzhou 450001, China.

Tel.: 0371-67758022; Fax: 0371-67758021

Supplemental Tables

Table S1. Candidate genes potentially related to dityrosine reduction in HNGD-XJ9.

Gene ID	Database/Category	Putative function
GE001281	eggNOG / [E] Amino acid transport and metabolism	Encodes an enzyme involved in aromatic amino acid metabolism, participating in tyrosine and phenylalanine catabolism [1,2]
GE001328	KEGG / Tyrosine metabolism pathway	Encodes homogentisate dioxygenase, capable of catalyzing oxidative ring cleavage of aromatic structures [3]
GE002892	KEGG / Aromatic compound degradation pathway	Encodes an aromatic ring dioxygenase that specifically catalyzes oxidative cleavage of aromatic conjugated systems [4,5]

Note: These genes were identified based on genomic annotation and are predicted to be involved in dityrosine reduction.

Table S2. Antibiotic resistance genes detected in the HNGD-XJ9 genome.

Gene ID	ARO_name	Resistance type	Resistance mechanism	Identity (%)	Coverage (%)
GE000031	mtrA	Macrolide antibiotic, penam	Antibiotic efflux	45.12	96.17
GE000032	arlS	Disinfecting agents and antiseptics	Antibiotic efflux	35.84	36.22
GE000043	FusF	Fusidic acid	Antibiotic target protection	41.09	93.02
GE000062	tetT	Tetracycline antibiotic	Antibiotic target protection	27.41	88.84

Note: The sequence homologies to known resistance genes were all below the 80% threshold, suggesting a low risk of horizontal gene transfer.

Table S3. Potential pathogenicity-related genes in HNGD-XJ9 based on PHI-base annotation.

Gene ID	Pathogen species	Identity (%)	Coverage (%)
GE000006	*Burkholderia glumae*	41.25	93.65
GE000008	*Xanthomonas oryzae*	30.93	47.00
GE000010	*Streptococcus pneumoniae*	32.83	98.21
GE000018	*Fusarium oxysporum*	26.42	61.51

Note: The sequence identities were all below the 80% identity threshold for virulence determinants, indicating no significant pathogenicity-related genes.

References

1. Arias-Barrau, E.; Olivera, E.R.; Luengo, J.M.; Fernández, C.; Galán, B.; García, J.L.; Díaz, E.; Miñambres, B. The homogentisate pathway: A central catabolic pathway involved in the degradation of L-phenylalanine, L-tyrosine, and 3-hydroxyphenylacetate in *Pseudomonas putida*. *J. Bacteriol*. **2004**, *186*, 5062-5077.
2. Moran, G.R. 4-Hydroxyphenylpyruvate dioxygenase. *Arch. Biochem. Biophys*. **2005**, *433*, 117-128.
3. Jeoung, J.H.; Bommer, M.; Lin, T.Y.; Dobbek, H. Visualizing the substrate-, superoxo-, alkylperoxo-, and product-bound states at the nonheme Fe(II) site of homogentisate dioxygenase. *Proc. Natl. Acad. Sci. USA*. **2013**, *110*, 12625-12630.
4. Seo, J.S.; Keum, Y.S.; Li, Q.X. Bacterial degradation of aromatic compounds. *Int. J. Environ. Res. Public Health*. **2009**, *6*, 278-309.
5. Liu, Y.; Yang, J.; Li, R.; Long, L. Research advances of microbial Rieske-type aromatic ring dioxygenases. *J. Guangxi Acad. Sci*. **2020**, *36*, 272-281. (in Chinese)
